# Temporal Dynamics of Phytochemicals in Selected Medicinal Plants Across Gandaki Province, Nepal

**DOI:** 10.1002/fsn3.71802

**Published:** 2026-04-29

**Authors:** Dipak Paudel, Santosh Koirala, Dhaka Ram Bhandari, Achyut Adhikari, Bhoj Raj Poudel, Megh Raj Pokhrel

**Affiliations:** ^1^ Central Department of Chemistry Tribhuvan University Kathmandu Nepal; ^2^ Department of Chemistry, Prithvi Narayan Campus Tribhuvan University Pokhara Nepal; ^3^ Center for Environmental and Sustainable Agriculture Research (CESAR) Pokhara Nepal; ^4^ Department of Chemistry, Tri‐Chandra Multiple Campus Tribhuvan University Kathmandu Nepal

**Keywords:** Antioxidants, *Cinnamomum tamala*, *Curcuma longa*, *Phytochemicals*, Time interval analysis, *Zanthoxylum armatum*

## Abstract

Many phytochemicals, including phenols and flavonoids, are valued for their anti‐inflammatory, antioxidant, antibacterial, and anti‐cancer properties. However, these compounds are susceptible to chemical degradation when exposed to heat, light, oxygen, or changes in pH. Understanding their stability over time is essential for ensuring the quality, safety, and efficacy of natural products. This study investigated temporal changes in phytochemical content and antioxidant activity in three commonly used medicinal plants in Nepal: *Cinnamomum tamala* ( *C. tamala* ) leaves, *Curcuma longa* ( *C. longa* ) rhizome, and *Zanthoxylum armatum* (*Z. armatum*) fruits. Standard Folin–Ciocalteu reagent method for TPC, AlCl_3_ colorimetric method for TFC, and DPPH free radical scavenging assay for antioxidant activity were used. Results showed a significant decline in total phenolic content (TPC), total flavonoid content (TFC), and antioxidant activity over the 1‐year storage period (*p* < 0.001). All three species showed decreases in TPC and TFC, but for *C. tamala* , the TFC reduction between 6 and 12 months was not significant (*p* = 0.0551), indicating partial stabilization. Antioxidant activity, measured as IC_50_, decreased over time, compared with standard ascorbic acid values, indicating reduced capacity. Most changes were statistically significant across time points. Analysis of altitude revealed no substantial effect on TPC, TFC, or IC_50_ for *C. tamala* and *Z. armatum*. Only *C. longa* exhibited a minor positive correlation between altitude and TFC (*p* = 0.045). In conclusion, storage duration markedly reduces the phytochemical content and antioxidant potential of these medicinal plants. These findings emphasize the importance of proper storage to preserve bioactive compounds and provide guidance for selecting high‐quality plant materials. The results are relevant for optimizing the use of natural antioxidants in foods, spices, nutraceuticals, and pharmaceutical products.

## Graphical abstract

A comparative phytochemical analysis of selected medicinal plants shows a decrease in antioxidant properties with temporal changes; furthermore, there is no significant relationship between antioxidant activity and geographical location.

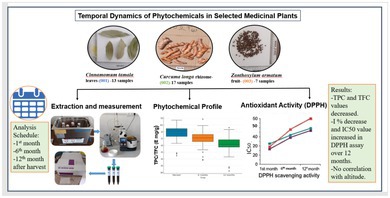

## 1 Introduction

Medicinal plants are vital to the healthcare industry worldwide, with approximately 10% of all vascular plants, roughly 35,000 to 50,000 species, possessing recognized medicinal properties (Salmerón‐Manzano et al. 2020). In Nepal, around 300 species are reported to have medicinal value (Pyakurel et al. 2019). Despite the availability of modern pharmaceuticals, not all patients benefit equally due to limitations in potency, side effects, and tolerance, highlighting the ongoing need to explore alternative or complementary therapeutic options (Asante et al. 2019). Bioactive substances called phytochemicals are included in foods and supplements derived from plants and are being used more and more to prevent disease and promote health. Functional foods contain phytochemicals in natural form, whereas nutraceuticals provide them in concentrated preparations (Kumar et al. 2023). Commonly found in fruits, vegetables, seeds, nuts, roots, and grains, these substances include alkaloids, phenolics, flavonoids, glycosides, lignins, and tannins. They function in a range of biological actions, including antibacterial, anti‐inflammatory, anti‐cancer, and antioxidant properties (Scalbert et al. 2005; Alemán et al. 2022). Since reactive oxygen species are constantly produced during regular physiological processes, phytochemicals' antioxidant qualities are especially important since they contribute to preventing oxidative stress. Cancer, autoimmune diseases, neurological and cardiovascular disorders, and aging‐related issues are among several diseases linked to an imbalance between the generation of reactive oxygen species and antioxidant defenses (Waris and Ahsan 2006).

Phenolic compounds constitute a diverse and widespread class of secondary metabolites produced via the phenylpropanoid and shikimate pathways. Their aromatic rings with hydroxyl groups enable them to act as effective electron donors, directly contributing to antioxidant activity (Robards et al. 1999; Bendary et al. 2013; Lee et al. 2015; Tungmunnithum et al. 2018). Flavonoids, a subclass of phenolics, also exhibit strong free radical scavenging capabilities. Total phenolic content (TPC), total flavonoid content (TFC), and free radical scavenging (DPPH assays) are widely employed to measure the antioxidant potential of plants, natural products, and extracts (Brighente et al. 2007). Numerous studies have quantified TPC and TFC in extracts from fresh plants, correlating these measures with antioxidant activity (Mehmood et al. 2022; Phuyal et al. 2020). Natural product‐based applications are advantageous because they can slow the oxidation of biomolecules, including proteins, lipids, and DNA, and preserve endogenous antioxidants even at low concentrations (Becker et al. 2004). However, phytochemicals are prone to degradation in response to environmental conditions, including light, heat, oxygen, pH variations, and oxidants, which can diminish their bioactivity over time (DeBenedictis et al. 2023; Jia et al. 2025).

The chemical and biological composition of plants is affected by a range of factors, including cultivation area, climate, growth age and stage, genetic variation, and environmental stressors (Hasan et al. 2024; Miliauskas et al. 2004; Mykhailenko et al. 2025). In Nepal, the rich diversity of medicinal flora, increasing export demand, traditional reliance on herbal remedies, and investments in research have promoted the exploration of bioactive plant compounds (Singh et al. 1979). Recent studies indicate that Nepali herbs and spices contain abundant natural antioxidants that often exceed the levels found in common dietary foods (Aryal et al. 2019; Kutal et al. 2021; Lamichhane et al. 2023; Shrivastava et al. 2023). Several herbs and spices, including *C. longa* roots (Hyaunmikha and Subba 2023), *Z. armatum* fruit (Pathak et al. 2021; Phuyal et al. 2020), *C. tamala* leaves (Tandukar et al. 2022), have been reported to contain flavonoids and exhibit antioxidant activity. However, results vary depending on geographic location, agricultural practices, extraction methods, and solvents, making direct comparisons challenging. While natural antioxidants are increasingly used as food additives to inactivate free radicals, data on the temporal stability of bioactivity like phenolic content, flavonoid content, and antioxidant activity under storage conditions are limited. Few studies have systematically assessed changes in these bioactive components over time.

To address these gaps, this study investigates the stability of total phenolic and total flavonoid content, as well as antioxidant activity in selected plant species from Gandaki Province, Nepal, over 1 year. Methanol was used as the extraction solvent, employing ultrasonic‐assisted extraction to ensure consistent recovery of phytochemicals. Samples were analyzed at 1, 6, and 12 months post‐harvest. Understanding the temporal dynamics of phytochemical content and antioxidant activity can inform the selection of high‐quality plant materials and guide the future development of natural antioxidants for use in food, nutraceutical, pharmaceutical, and therapeutic applications.

## 2 Materials and Methods

### 2.1 Chemicals and Reagents

Analytical grade chemicals were used in this study. 2,2‐diphenyl‐1‐picrylhydrazyl (DPPH, ≥ 99%), Folin–Ciocalteau (FC) reagent, and anhydrous aluminum chloride were purchased from Sigma‐Aldrich (Mumbai, India). Gallic acid was purchased from Hi‐Media (India), while methanol, anhydrous sodium carbonate, and sodium nitrite were procured from Fisher Scientific (India).

### 2.2 Sample Collection and Extraction

Herbs and spices ( *Cinnamomum tamala* (001)‐13 samples, *Curcuma longa* (002)‐17 samples and *Zanthoxylum armatum* (003)‐7 samples) were collected from multiple locations within Nepal's Gandaki region, accounting for potential geographical variation during a single season (winter) between December 2022 and January 2023. Plant parts were thoroughly cleaned with water when necessary and then dried in the shade for 7 days at room temperature, with temperature and humidity monitored to avoid direct sunlight exposure. Once dried, the plant material was crushed using a Philips HL7759/00 Mixer Grinder (750 W). The powdered plant material was extracted using polar solvent, methanol in a 1:10 solute‐to‐solvent ratio via ultrasonic‐assisted extraction (UAE) (Sonicator bath‐UC‐30A, Biobase Industry, Shandong, China; 50 Hz, 220 V) at 20°C for 30 min (Kumar et al. 2020; Shen et al. 2023). A rotary evaporator was used to concentrate the extracts under reduced pressure until a solid mass was achieved and then freeze‐dried for 1 day. Extracts were kept in sealed containers at 4°C until further analysis. Extraction yields were calculated to standardize sample preparation. Plants and their taxonomic identification were done at the National Herbarium in Godavari, Nepal.

Plant samples were kept in sealed plastic zipper bags at room temperature for time‐interval analysis, within a wooden cupboard, away from direct sunlight, with continuous monitoring of temperature and humidity. At each designated time point, the stored samples were subjected to the same extraction protocol to ensure consistency in comparative analyses.

### 2.3 Bioactivity Assay

#### 2.3.1 TPC

The Folin–Ciocalteu (FC) reagent method, as explained by Li et al. (2013), was used to determine the plant extracts' total phenolic content (TPC), with slight modifications (Li et al. 2013). Gallic acid was used to generate a standard calibration curve, and TPC values were reported in milligrams of gallic acid equivalents per gram of dry extract (mg GAE/g). For calibration, gallic acid solutions in 400 μL were prepared with concentrations of 10, 20, 30, 40, 50, 60, 70, 80, 90, and 100 μg/mL. For sample analysis, 400 μL of plant methanolic extract was added to 1600 μL of 10% sodium carbonate solution, followed by the mixing of 2000 μL of FC reagent. For 1 h, the mixture endured incubation at room temperature. Using the same process, a blank solution was prepared with methanol in place of the plant extract. A UV–visible spectrophotometer (Biobase Bioyu Co. Ltd., China; λ range 200–800 nm) was used to record the absorbance at 760 nm, and all measurements were made in triplicate. The standard curve of gallic acid was generated using the same procedure to calculate the TPC of the samples.

The following formula was used to determine the TPC:

(1) C=cV/m

Here, C = TPC in mg GAE/g, c = Gallic acid content determined by the calibration curve (mg/mL), V = volume of plant extract (mL), and m = weight in taken volume of the plant extract (g).

All results were expressed as median (Q1–Q3). For the time‐interval analysis, the same extraction and TPC determination protocol was applied to samples stored for 6 and 12 months after harvest.

#### 2.3.2 TFC

With some slight adjustments, the AlCl_3_ colorimetric method, as described by Shraim et al. (2021), was used to quantify the total flavonoid content (TFC) of the plant extracts (Shraim et al. 2021). Standard solutions of quercetin have been prepared with concentrations varying from 0 to 1000 μg/mL for calibration. For analysis of TFC, 500 μL of plant extract was mixed with 0.5 M aqueous sodium nitrite of 150 μL and 0.3 M AlCl_3_ of 150 μL in a test tube. After 6 min, 1 M sodium hydroxide of 1 mL was added, and also 3.2 mL of distilled water was added. After thoroughly mixing the mixture, a UV–visible spectrophotometer (Biobase Bioyu Co. Ltd., China) was used to measure the absorbance at 510 nm in a 96‐well plate. A blank was prepared using methanol in place of the extract. Standard quercetin solutions were measured in a similar manner to generate the calibration curve. Every experiment was carried out in triplicate, and the median (Q1‐Q3) was used to express the results. Quercetin calibration curve equation was use for calculation of TFC and milligrams of quercetin equivalent per gram of dry extract (mg QE/g) as expressed unit.

The total flavonoid content (TFC) was calculated using Equation 1, where C represents the TFC expressed as mg Quercetin Equivalents per gram (mg QE/g) of extract. In this equation, c is the quercetin concentration (mg/mL) determined by the calibration curve, V is the total volume of the extract in mL, and m is the mass of the extract used in the taken volume of the plant extract (g).

#### 2.3.3 DPPH Assay

The free radical scavenging activity (DPPH assay) was performed for all extracts using the standard method described by Phuyal et al. (2020) with minor modifications, and for reference standard ascorbic acid was used (Phuyal et al. 2020). The extracts' stock solutions and ascorbic acid at concentrations of 25, 50, 75, 100, 125, and 150 μg/mL were prepared in methanol. For the assay, methanolic 1 mL of 0.002% DPPH solution was mixed with 1 mL of each varied concentration extract in separate test tubes. The mixes were left to incubate in the dark at room temperature for thirty minutes. After incubation, 200 μL aliquots were transferred in triplicate into the wells of a 96‐well plate. At 517 nm, absorbance was measured using a UV–Vis spectrophotometer (Biobase Bioyu Co. Ltd., China). 1 mL methanol with 1 mL DPPH solution was also measured for a control condition. Every experiment was carried out in triplicate, and the mean ± SD was used to express the results. The scavenging activity of each extract was calculated using the standard formula based on control and sample absorbance values.

The following formula was used to determine the percentage of DPPH radical scavenging activity (I%):

(2) $$I\%=\left[\left(A\_\text{control}-A\_\text{sample}\right)/A\_\text{control}\right]\times 100$$

Here, A_control = absorbance of the methanol and DPPH solution and A_sample = absorbance of the extract or standard with DPPH solution.

The extracts' IC_50_ values were used to express the percentage of inhibition (I%). The amount of plant extract needed to scavenge 50% of DPPH free radicals is known as the median inhibitory concentration, or IC_50_. By graph plotting the percentage of inhibition against the extract concentration, the IC_50_ value for each sample was determined. Calculations were performed using GraphPad Prism 9 software, employing the inhibitor versus normalized response—variable slope least squares fitting method.

### 2.4 Statistical Analysis

All analyses were carried out in Python using the pandas and statsmodels libraries. The figures were compiled in Python using the matplotlib and seaborn libraries. The impact of storage duration on IC_50_ values, total phenolic content (TPC), and total flavonoid content (TFC) was compared using a one‐way analysis of variance (ANOVA) for each variable; the overall ANOVA test was set at *p* < 0.05 for statistical significance.

A post hoc Tukey's Honestly Significant Difference (HSD) test was used to assess pairwise comparisons between storage times when the ANOVA revealed significant differences between groups. The Tukey HSD test controlled for multiple comparisons, and statistical significance for the adjusted *p*‐values was also set at *p* < 0.05. Results are summarized as median with interquartile range (Q1–Q3), and significant groupwise differences are indicated with lowercase letters in the figures.

To evaluate the relationship between phytochemical content and altitude, multiple linear regression analyses were performed. Total phenolic content (TPC), total flavonoid content (TFC), and IC_50_ values were each modeled separately as dependent variables, with altitude as the independent variable. Model coefficients, *R* ^2^ values, and *p*‐values (statistical significance was set at *p* < 0.05) were reported to assess the strength and significance of the associations.

## 3 Results

### 3.1 TPC Declines Over Storage Time in All Species

**FIGURE 1:**
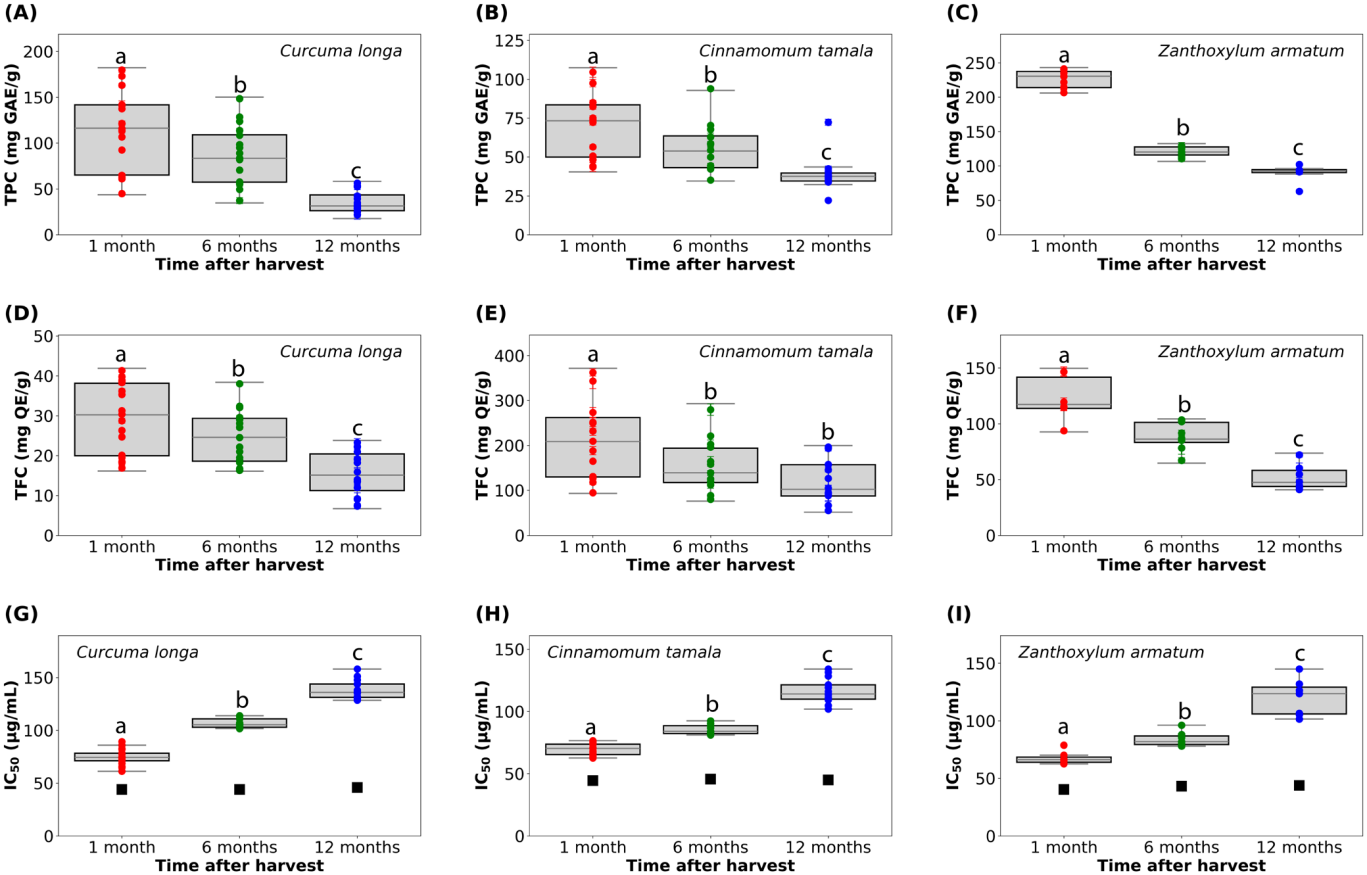
The distribution of TPC (1 (A–C)); TFC (1 (D–F)); and IC_50_ (1 (G–I)) values across different storage times for *C. tamala* , *C. longa* , an*d Z. armatum*, respectively. Black dot of 1 (G–I) are IC_50_ of ascorbic acid.

Standard Gallic acid calibration curve, as shown in Figure S1 was used for calculate total phenolic content (TPC). The distribution of TPC values across different storage times is shown in Figure 1A–C as box plots overlaid with individual sample points. Median (Q1–Q3) values are summarized in Table 1. The detailed data are in Table S1A–C.

**TABLE 1:** Summary table of TPC for a year in the given samples.

Sample	TPC in 1st month (mg GAE/g), mean (Q1–Q3)	TPC in the 6th month (mg GAE/g), mean (Q1–Q3)	TPC in 12th month (mg GAE/g), mean (Q1–Q3)
*C. tamala* (001)	73.18 (49.91–83.46)	53.73 (43.05–63.50)	37.50 (34.47–39.71)
*C. longa* (002)	116.12 (65.13–141.62)	116.12 (65.13–141.62)	31.37 (26.13–43.38)
*Z. armatum* (003)	230.53 (213.82–237.58)	120.09 (116.02–127.70)	93.11 (90.37–94.60)

Tukey HSD results are indicated by different letters above the boxes in the plot (Figure 1): significant differences exist between time points with distinct letters, but not between those that share the same letter. For *Cinnamon tamala*, the median TPC at 1 month was 73.18 (49.91–83.46) mg QE/g, which decreased to 53.73 (43.05–63.50) mg QE/g by 6 months and further declined to 37.50 (34.47–39.71) mg QE/gat 12 months. *Curcuma longa* showed a median TPC of 116.12 (65.13–141.62) mg QE/g at 1 month, decreasing to 83.38 (57.25–109.00) mg QE/g at 6 months and 31.37 (26.13–43.38) mg QE/g at 12 months. *Zanthoxylum aramatum* had the highest initial TPC, 230.53 (213.82–237.58) mg QE/g at 1 month, which declined to 120.09 (116.02–127.70) mg QE/g at 6 months and 93.11 (90.37–94.60) mg QE/g at 12 months.

One‐way ANOVA indicated highly significant differences in TPC across storage times for all extracts ( *C. tamala* : *p* = 5.60 × 10^−12^; *C. longa* : *p* = 6.13 × 10^−25^; *Z. aramatum*: *p* = 3.71 × 10^−45^). Post hoc Tukey HSD tests confirmed that all pairwise comparisons between 1, 6, and 12 months were significant for each extract (*p* < 0.01).

### 3.2 TFC Declines Over Storage Time in All Species

As seen in Figure S2, the total flavonoid content (TFC) was determined using a standard quercetin calibration curve. The distribution of TFC values across different storage times is shown in Figure 1D–F as box plots overlaid with individual sample points. Median (Q1–Q3) values are shown in Table 2.

**TABLE 2:** TFC summary table for a year in the given samples.

Sample	TFC in 1st month (mg QE/g), mean (Q1–Q3)	TFC in 6th month (mg QE/g), mean (Q1–Q3)	TFC in 12th month (mg QE/g), mean (Q1–Q3)
*C. tamala* (001)	208.75 (130.00–262.25)	139.25 (117.25–193.95)	102.30 (87.50–157.00)
*C. longa* (002)	30.23 (19.98–38.14)	24.56 (18.61–29.33)	15.10 (11.19–20.39)
*Z. armatum* (003)	117.25 (113.75–141.75)	86.25 (83.25–101.25)	47.35 (43.75–58.25)

Tukey HSD results are indicated by different letters above the boxes in the plot (Figure 1): significant differences exist between time points with distinct letters, but not between those that share the same letter. For *C. tamala* , median TFC was 208.75 (130.00–262.25) mg QE/g at 1 month, decreasing to 139.25 (117.25–193.95) mg QE/g at 6 months and 102.30 (87.50–157.00) mg QE/g at 12 months. *Curcuma longa* extract showed 30.23 (19.98–38.14) mg QE/gat 1 month, 24.56 (18.61–29.33) mg QE/g at 6 months, and 15.10 (11.19–20.39) at 12 months. *Zanthoxylum aramatum* had median TFC values of 117.25 (113.75–141.75) mg QE/g, 86.25 (83.25–101.25) mg QE/g, and 47.35 (43.75–58.25) mg QE/g at 1, 6, and 12 months, respectively.

One‐way ANOVA demonstrated significant differences among storage times for all extracts ( *C. tamala* : *p* = 2.78 × 10^−8^; *C. longa* : *p* = 2.85 × 10^−18^; *Z. aramatum*: *p* = 2.02 × 10^−22^). Tukey HSD analysis indicated that for *C. tamala* , TFC differed significantly between 1 and 6 months (*p* = 0.0004) and 1 and 12 months (*p* < 0.001); however, there was no significant difference between 6 and 12 months (*p* = 0.0551), suggesting stabilization after 6 months. For *C. longa* and *Z. aramatum*, all pairwise comparisons were significant (*p* < 0.01), indicating a continuous decline over the 12 months.

### 3.3 Antioxidant Potential Decreases Over Storage as Indicated by Increasing IC_50_

Using the DPPH free radical scavenging experiment, the three species' antioxidant capacity was assessed, and its reducing power was calculated using the 50% inhibitory concentration (IC_50_). Ascorbic acid was used as the standard. Figure 1G–I present IC_50_ values across storage times, with median (Q1–Q3) values shown in Table 3.

**TABLE 3:** Summary table of IC_50_ for a year in the given samples.

Sample	IC_50_ in 1st month (μg/mL), mean (Q1–Q3)	IC_50_ in 6th month (μgmg/mL), mean (Q1–Q3)	IC_50_ in 12th month (μg/mL), mean (Q1–Q3)
Ascorbic acid	40.35–44.46	43.16–45.64	43.92–45.89
*C. tamala* (001)	70.32 (65.49–73.77)	83.99 (82.21–88.54)	114.02 (109.93–121.44)
*C. longa* (002)	74.37 (71.15–78.30)	105.33 (102.80–110.78)	135.95 (131.22–143.88)
*Z. armatum* (003)	66.24 (64.03–68.50)	81.99 (79.42–86.84)	123.65 (105.99–129.17)

Tukey HSD results are indicated by different letters above the boxes in the plot (Figure 1): significant differences exist between time points with distinct letters, but not between those that share the same letter. The time interval analysis I% for each sample is given in the Table S2. As shown by the black square markers in Figure 1G–I, all data were compared to the standard ascorbic acid's IC_50_ value, which ranged from 40.35 to 44.46 μg/mL.

For *C. tamala* , median IC_50_ increased from 70.32 (65.49–73.77) μg/mL at 1 month to 83.99 (82.21–88.54) μg/mL at 6 months and 114.02 (109.93–121.44) μg/mL at 12 months. *Curcuma longa* extract showed an increase from 74.37 (71.15–78.30) μg/mL at 1 month to 105.33 (102.80–110.78) μg/mL at 6 months and 135.95 (131.22–143.88) μg/mL at 12 months. *Zanthoxylum aramatum* had median IC_50_ values of 66.24 (64.03–68.50) μg/mL, 81.99 (79.42–86.84) μg/mL, and 123.65 (105.99–129.17) μg/mL at 1, 6, and 12 months, respectively.

ANOVA confirmed highly significant differences across storage times for all extracts ( *C. tamala* : *p* = 1.31 × 10^−18^; *C. longa* : *p* = 3.06 × 10^−29^; *Z. aramatum*: *p* = 9.24 × 10^−8^). Tukey HSD analysis indicated significant differences among all pairwise comparisons for *C. tamala* and *C. longa* (*p* < 0.001). For *Z. aramatum*, all comparisons were significant, although the difference between 1 and 6 months was less pronounced (*p* = 0.0228). The time and variable (bioactivities) *p* value are in Tables S3 and S4.

### 3.4 Effect of Altitude on Phytochemical Content and Antioxidant Activity

**FIGURE 2:**
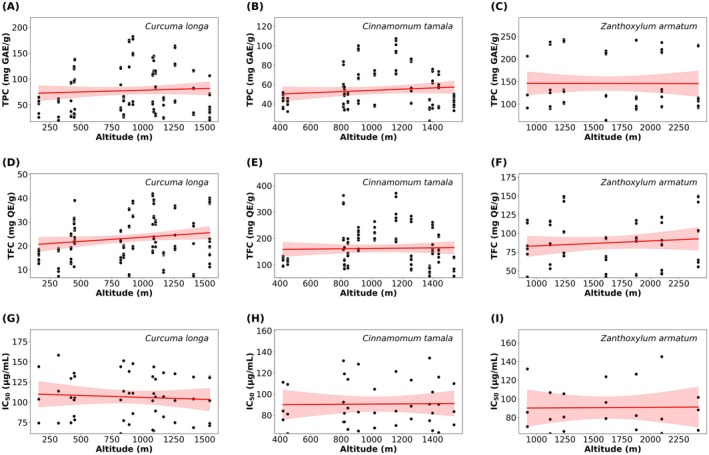
Relationship between altitude and phytochemical parameters (TPC, TFC, and IC_50_). Scatterplots show total phenolic content (TPC, mg GAE/g), total flavonoid content (TFC, mg QE/g), and antioxidant activity (IC_50_, μg/mL) as a function of sampling altitude for *Curcuma longa* , *Cinnamomum tamala* , and *Zanthoxylum armatum*. Black points represent individual sample measurements. Red lines indicate fitted regression models, and shaded bands represent the 95% confidence intervals for the predicted means. *Note*: *The distribution of TPC (1 (A–C)); TFC (1 (D–F)); and IC_50_ (1 (G–I)) values across different altitude for *C. longa*, *C. tamala*, and *Z. armatum*, respectively.

To examine the impact of altitude on phytochemical content and antioxidant activity, multiple linear regression was performed with TPC, TFC, and IC_50_ as dependent variables and altitude (m) as the independent variable shown in Figure 2.

The influence of altitude on total phenolic content (TPC), total flavonoid content (TFC), and IC_50_ of antioxidant activity for *Cinnamomum tamala* , *Curcuma longa* , and *Zanthoxylum aramatum* is shown in Table 4.

**TABLE 4:** Effect of altitude on TPC, TFC, and IC_50_ by multiple linear regression.

Sample	Dependent variables	Independent variables	Regression coefficient	*R* ^2^	*p*
*C. tamala* (001)‐TPC	TPC (mg GAE/g)	Altitude (m)	0.006291	0.012511	0.229893
*C. tamala* (001)‐TFC	TFC (mg QE/g)	Altitude (m)	0.006076	0.000858	0.753951
*C. tamala* (001)‐IC_50_	IC_50_ (μg/mL)	Altitude (m)	0.000957	0.000269	0.921126
*C. longa* (002)‐ TPC	TPC (mg GAE/g)	Altitude (m)	0.006721	0.004059	0.433991
*C. longa* (002) ‐TFC	TFC (mg QE/g)	Altitude (m)	0.003507	0.026236	0.045466
*C. longa* (002)‐IC_50_	IC_50_ (μg/mL)	Altitude (m)	−0.004776	0.005482	0.605595
*Z. armatum* (003)‐TPC	TPC (mg GAE/g)	Altitude (m)	−0.000447	0.000015	0.976081
*Z. armatum* (003)‐TFC	TFC (mg QE/g)	Altitude (m)	0.006619	0.011446	0.403967
*Z. armatum* (003)‐IC_50_	IC_50_ (μg/mL)	Altitude (m)	0.000711	0.000228	0.948188

*Note:* *p*‐value is > 0.05 for all others. Altitude has no effect on TPC, TFC, or IC_50_ except for *C. longa* ‐TFC.

For *C. tamala* , altitude did not significantly influence TPC (*β* = 0.0063, *R* ^2^ = 0.0125, *p* = 0.230), TFC (*β* = 0.0061, *R* ^2^ = 0.0009, *p* = 0.754), or IC_50_ (*β* = 0.0010, *R* ^2^ = 0.0003, *p* = 0.921). Similarly, in *Z. armatum*, no significant associations were observed for TPC (*β* = −0.0004, *R* ^2^ = 0.00002, *p* = 0.976), TFC (*β* = 0.0066, *R* ^2^ = 0.0114, *p* = 0.404), or IC_50_ (*β* = 0.0007, *R* ^2^ = 0.0002, *p* = 0.948).

In contrast, for *C. longa* , altitude showed a minor positive relationship with TFC (*β* = 0.0035, *R* ^2^ = 0.0262, *p* = 0.045), indicating that higher altitudes were associated with increased flavonoid content. No significant effects of altitude were observed on TPC (*β* = 0.0067, *R* ^2^ = 0.0041, *p* = 0.434) or IC_50_ (*β* = −0.0048, *R* ^2^ = 0.0055, *p* = 0.606).

Overall, multiple linear regression analysis revealed a mixed effect of altitude, with only *C. longa* showing a very minor increase in flavonoid content at higher elevations. Other species and parameters remained unaffected by altitude.

## 4 Discussion

### 4.1 Declines in TPC, TFC, and Antioxidant Activity With Storage Time

This study's principal goal was to evaluate the stability of basic phytochemical properties including total phenolic content (TPC), total flavonoid content (TFC), and antioxidant activity (IC_50_) in the methanolic extracts of *Cinnamomum tamala* , *Curcuma longa* , and *Zanthoxylum aramatum* over 12 months. Across all three species, we observed a consistent decline in TPC and TFC as storage time increased, which was accompanied by a corresponding decrease in antioxidant potential. This trend aligns with previous studies, which have demonstrated that phenolic and flavonoid compounds are exceptionally vulnerable to decline during storage due to oxidation, polymerization, and enzymatic or chemical transformations (Singh and Madan 2018; Yap et al. 2023).

The observed decreases were particularly pronounced between fresh samples and those stored for 6 or 12 months, indicating a strong time‐dependent effect on phytochemical stability. Factors influencing this degradation include light, heat, temperature moisture content, and packaging materials, all of which can accelerate oxidative reactions and reduce the bioavailability of active compounds (Souza et al. 2013; Lisboa et al. 2018; Dehel Gamage et al. 2021). Specifically, the decline in TPC and TFC in *C. tamala* , *C. longa* , and Z. *aramatum* highlights the importance of optimized storage conditions in preserving the pharmacological potential of plant‐derived extracts (Ali et al. 2018; Rahim et al. 2020; Sravanthi 2025). The stability of secondary metabolites is considered to be affected by a combination of intrinsic and extrinsic four factors, including genetic variation, ontogenetic stage, and environmental conditions (Akula and Ravishankar 2011; Koricheva and Barton 2012; Verma and Shukla 2015). In our study, differences in initial TPC and TFC among fresh samples of the identical species that were collected from different regions suggest that environmental factors, such as soil composition, climate, and seasonal variation, contribute to baseline phytochemical content. This variability is consistent with prior reports that secondary metabolites are inherently dynamic and responsive to both abiotic and biotic stressors, including temperature fluctuations, light intensity, UV exposure, water availability, nutrient deficits, and plant developmental stage (Gobbo Neto et al. 2007; Akula and Ravishankar 2011; Verma and Shukla 2015).

### 4.2 Antioxidant Activity and IC_50_ Trends

The antioxidant potential, measured as IC_50_ in DPPH assays, increased over the storage period for all three species, reflecting a reduction in free radical scavenging capacity. Fresh extracts displayed higher antioxidant activity, consistent with their elevated TPC and TFC levels. These findings align with the well‐established correlation between antioxidant activity and phenolic content, where the degradation of phenolics directly leads to a diminished radical‐scavenging capacity (Kim et al. 2019; Plaskova and Mlcek 2023). The inverse relationship between IC_50_ and antioxidant strength is a fundamental characteristic of the DPPH assay, where higher IC_50_ values indicate weaker activity (Zheng et al. 2010; Martinez‐Morales et al. 2020). Reference standard ascorbic acid was used in this study, providing a benchmark to evaluate extract potency. However, none of the plant extracts matched the potency of ascorbic acid; the relative decreases over time were consistent across all species.

Among the three species, Z. *armatum* demonstrated the highest initial TPC and TFC, followed by *C. longa* and *C. tamala* , which aligns with earlier research (Phuyal et al. 2020; Tandukar et al. 2022). Methanol's strong polarity led to its use as the extraction solvent in this investigation, which efficiently solubilizes phenolics, flavonoids, and other bioactive metabolites (Stalikas 2007; Do et al. 2014). Differences between our measured values and those previously published can be ascribed to elements such as the extraction method, sample processing, plant maturity, and environmental conditions.

### 4.3 Effects of Altitude

In contrast to the pronounced storage effects, altitude had a minimal impact on TPC, TFC, and IC_50_, except a modest positive correlation between altitude and TFC in *C. longa* . This finding suggests that postharvest and storage factors exert more substantial influence on phytochemical stability than initial environmental conditions. While some studies have reported increased phenolic or flavonoid accumulation at higher elevations due to UV‐B exposure or cooler temperatures (Gil et al. 2013; Santin et al. 2018), our findings indicate that the dominant influence of storage conditions may overshadow these altitude effects. A limited altitude range, sample size, and co‐variation with other environmental factors may further explain the lack of an apparent altitude effect.

### 4.4 Implications for Herbal Product Quality and Standardization

The decrease of phytochemicals and associated fall in antioxidant activity over storage have important implications for the pharmaceutical and nutraceutical industries. Variability in secondary metabolite content challenges the standardization of herbal extracts, underscoring the need for controlled storage conditions, rigorous quality control, and stability testing (Tanko et al. 2005; Efferth 2012; Booker et al. 2015; Abraham et al. 2023). The adoption of advanced analytical approaches, including chromatography‐mass spectrometry and untargeted metabolomics, allows for comprehensive profiling of bioactive compounds, detection of chemical shifts, and identification of chemotypes specific to geographical regions (Betz et al. 2011; Steinmann and Ganzera 2011; Brinckmann 2015; van Wyk and Prinsloo 2020).

Furthermore, artificial intelligence and machine learning approaches can aid in predicting biological activity, optimizing extraction protocols, and enhancing reproducibility in natural product research (Varghese et al. 2025), These strategies collectively enable the development of robust quality assurance frameworks that ensure the therapeutic effectiveness and safety of herbal remedies throughout production, storage, and distribution.

## 5 Conclusions

Overall, our study demonstrates a significant, storage‐driven decline in TPC, TFC, and antioxidant activity of *C. tamala* leaves, *C. longa* rhizome, and Z. *armatum* fruit over a 12‐month period. Geographical altitude played a minor role, with only a slight positive influence on *C. longa* TFC. These findings emphasize the vital significance of appropriate storage, standardized extraction protocols, and rigorous quality control measures in maintaining the bioactivity of plant‐derived products. Future studies should explore larger altitudinal gradients, integrate multi‐factorial environmental analyses, and assess fresh plant material across locations to better isolate the effects of geography from storage‐driven degradation.

## Author Contributions

**Dipak Paudel:** methodology, formal analysis, investigation, writing – original draft, data curation, validation. **Megh Raj Pokhrel:** supervision, writing – review and editing, conceptualization. **Achyut Adhikari:** conceptualization, methodology, writing – review and editing. **Bhoj Raj Poudel:** methodology, writing – review and editing. **Santosh Koirala:** data curation, investigation, validation, writing – original draft, visualization. **Dhaka Ram Bhandari:** conceptualization, methodology, visualization, supervision, writing – review and editing.

## Conflicts of Interest

The authors declare no conflicts of interest.

## Supporting information


**Figure S1:** Calibration curve of Gallic acid.
**Figure S2:** Calibration curve of Quercetin.
**Table S1:** (A). Analysis time, TPC, TFC, and IC_50_ values of 001—*Cinamomum tamala*‐13 samples.
**Table S1:** (B) Analysis time, TPC, TFC and IC_50_ values of 002— *Curcuma longa* ‐17 samples.
**Table S1:** (C). Analysis time, TPC, TFC, and IC_50_ values of 003—*Zanthoxylum armatum*‐7 samples.
**Table S2:** (A)‐1. Analysis time and I% value of 001—*Cinamomum tamala*‐13 samples (1st month‐1st Analysis).
**Table S2:** (A)‐2. Analysis time and I% value of 001—*Cinamomum tamala*‐13 samples (6th month‐2nd Analysis).
**Table S2:** (A)‐3 Analysis time and I% value of 001—*Cinamomum tamala*‐13 samples (12th month‐3rd Analysis).
**Table S2:** (B)‐1. Analysis time and I% value of 002— *Curcuma longa* ‐17 samples (1st month‐1st Analysis).
**Table S2:** (B)‐2. Analysis time and I% value of 002— *Curcuma longa* ‐17 samples (6th month‐2nd Analysis).
**Table S2:** (B)‐3. Analysis time and I% value of 002— *Curcuma longa* ‐17 samples (12th month‐3rd Analysis).
**Table S2:** (C)‐1. Analysis Time and I% value of 003‐*Zanthoxylum armatum*‐7 samples (1st month‐1st Analysis).
**Table S2:** (C)‐2. Analysis time and I% value of 003‐*Zanthoxylum armatum*‐7 samples (6th month‐2nd Analysis).
**Table S2:** (C)‐3. Analysis time and I% value of 003‐*Zanthoxylum armatum*‐7 samples (12th month‐3rd Analysis).
**Table S3:** Time and variable (phytochemical and antioxidant bioactivities)—*p*‐value.
**Table S4:** Time and variable (phytochemical and antioxidant bioactivities)—Group‐wise Tukey HSD summary.

## Data Availability

The data that support the findings of this study are available on request from the corresponding author. The data are not publicly available due to privacy or ethical restrictions.
