# Supplementary material for: Temporal Dynamics of Phytochemicals in Selected Medicinal Plants Across Gandaki Province, Nepal
Source: Food Sci Nutr. 2026 Apr 29;14(5):e71802. doi: 10.1002/fsn3.71802 (PMC13126094; doi:10.1002/fsn3.71802)
Supplement: Supplementary file 1 — Figure S1: Calibration curve of Gallic acid. Figure S2: Calibration curve of Quercetin. Table S1: (A). Analysis time, TPC, TFC, and IC50 values of 001—Cinamomum tamala‐13 samples. Table S1: (B) Analysis time, TPC, TFC and IC50 values of 002— Curcuma longa ‐17 samples. Table S1: (C). Analysis time, TPC, TFC, and IC50 values of 003—Zanthoxylum armatum‐7 samples. Table S2: (A)‐1. Analysis time and I% value of 001—Cinamomum tamala‐13 samples (1st month‐1st Analysis). Table S2: (A)‐2. Analysis time and I% value of 001—Cinamomum tamala‐13 samples (6th month‐2nd Analysis). Table S2: (A)‐3 Analysis time and I% value of 001—Cinamomum tamala‐13 samples (12th month‐3rd Analysis). Table S2: (B)‐1. Analysis time and I% value of 002— Curcuma longa ‐17 samples (1st month‐1st Analysis). Table S2: (B)‐2. Analysis time and I% value of 002— Curcuma longa ‐17 samples (6th month‐2nd Analysis). Table S2: (B)‐3. Analysis time and I% value of 002— Curcuma longa ‐17 samples (12th month‐3rd Analysis). Table S2: (C)‐1. Analysis Time and I% value of 003‐Zanthoxylum armatum‐7 samples (1st month‐1st Analysis). Table S2: (C)‐2. Analysis time and I% value of 003‐Zanthoxylum armatum‐7 samples (6th month‐2nd Analysis). Table S2: (C)‐3. Analysis time and I% value of 003‐Zanthoxylum armatum‐7 samples (12th month‐3rd Analysis). Table S3: Time and variable (phytochemical and antioxidant bioactivities)—p‐value. Table S4: Time and variable (phytochemical and antioxidant bioactivities)—Group‐wise Tukey HSD summary. [file FSN3-14-e71802-s001.docx]

**Supplementary materials**

**Supplementary Figures**

Figure S1: Calibration curve of Gallic acid


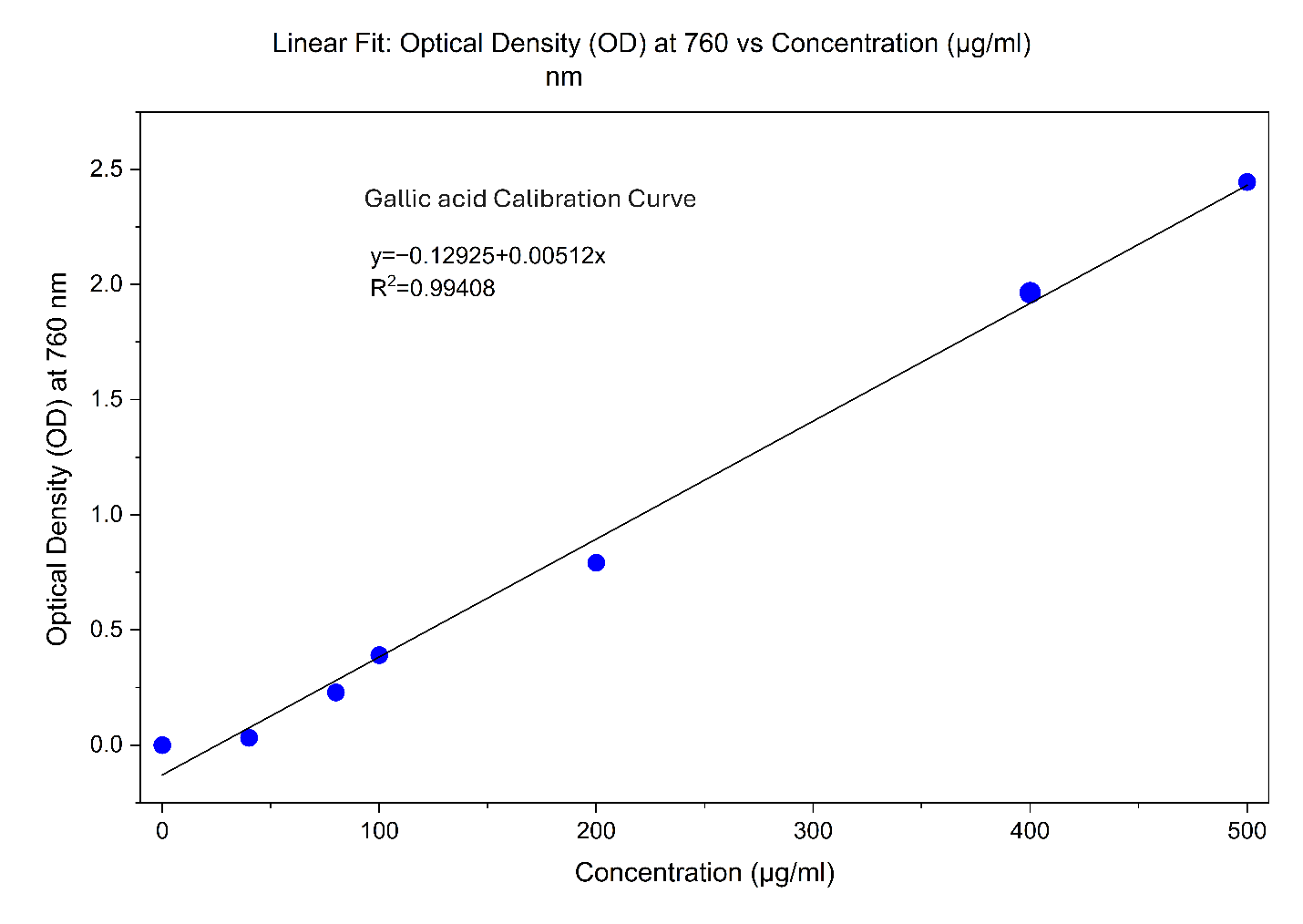


Figure S2: Calibration curve of Gallic acid


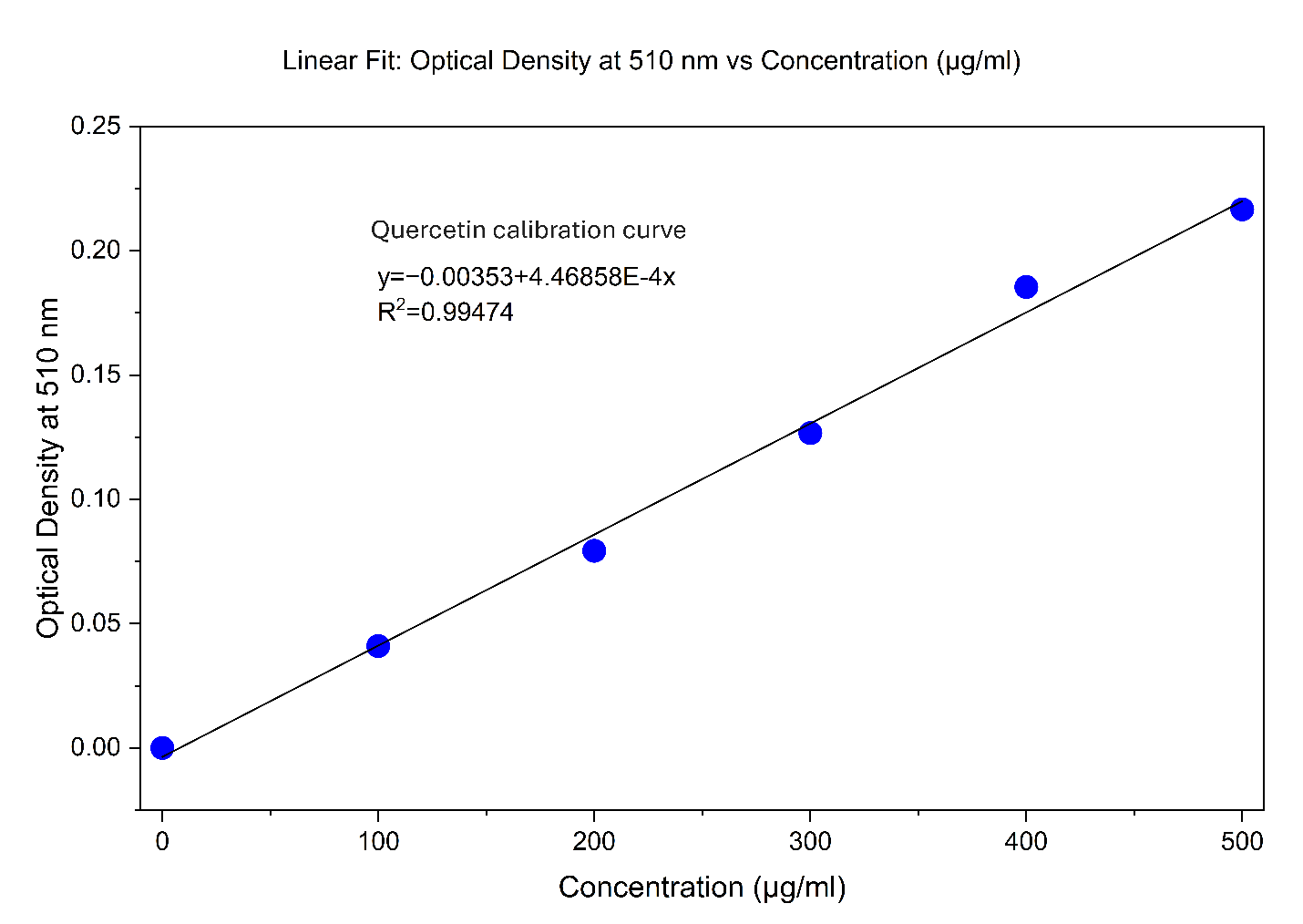


**Supplementary Tables**

Table S1: (A). Analysis time, TPC, TFC and IC50 values of 001- *Cinamomum tamala*-13 samples

| **Sample ID** | **Altitude(m)** | **Latitude** | **Longitude** | **Time** | **TPC (mg**  **GAE/g)**  **( 3 replicate)** | **TFC (mg QE/g)**  **( 3 replicate)** | **IC _50_ (µg/mL)** |
| --- | --- | --- | --- | --- | --- | --- | --- |
| 001A | 1540 | 28.1875 | 83.8424 | 1months | 48.3±1.76 | 127.25±2.17 | 70.95 |
| 001A | 1540 | 28.1875 | 83.8424 | 6 months | 42.76±0.84 | 79.91±4.04 | 83.47 |
| 001A | 1540 | 28.1875 | 83.8424 | 12 months | 36.28±2.95 | 54.91±3.21 | 109.93 |
| 001B | 1400 | 28.168 | 83.7679 | 1months | 75.13±1.43 | 251.58±9.75 | 65.49 |
| 001B | 1400 | 28.168 | 83.7679 | 6 months | 62.84±1.17 | 164.25±11.05 | 82.07 |
| 001B | 1400 | 28.168 | 83.7679 | 12 months | 37.78±1.98 | 145.75±4.92 | 101.93 |
| 001C | 816 | 28.0372 | 84.0803 | 1months | 82.47±1.81 | 343.58±17.21 | 73.699 |
| 001C | 816 | 28.0372 | 84.0803 | 6 months | 58.6±1.44 | 196.08±4.53 | 92.46 |
| 001C | 816 | 28.0372 | 84.0803 | 12 months | 38.58±1.79 | 157.75±1.32 | 131.51 |
| 001D | 1020 | 28.0679 | 84.5302 | 1 months | 73.98±0.52 | 249.91±12.42 | 68.06 |
| 001D | 1020 | 28.0679 | 84.5302 | 6 months | 70.29±0.88 | 221.25±1.32 | 82.21 |
| 001D | 1020 | 28.0679 | 84.5302 | 12 months | 38.12±0.71 | 196.43±3.48 | 104.7 |
| 001E | 822 | 28.1977 | 83.9729 | 1months | 56.43±1.2 | 164.91±2.02 | 73.77 |
| 001E | 822 | 28.1977 | 83.9729 | 6 months | 49.87±1.11 | 119.08±2.25 | 81.92 |
| 001E | 822 | 28.1977 | 83.9729 | 12 months | 33.92±0.54 | 93.75±8.52 | 119.21 |
| 001F | 1260 | 28.1095 | 83.8313 | 1months | 85.04±1.43 | 273.75±10.75 | 76.58 |
| 001F | 1260 | 28.1095 | 83.8313 | 6 months | 54.53±1.69 | 125.75±7.36 | 88.54 |
| 001F | 1260 | 28.1095 | 83.8313 | 12 months | 40.72±0.39 | 88.25±11.05 | 113.31 |
| 001G | 450 | 28.0838 | 84.2396 | 1months | 43.52±2.78 | 118.08±4.93 | 62.65 |
| 001G | 450 | 28.0838 | 84.2396 | 6 months | 42.79±0.46 | 112.25±7.36 | 81.11 |
| 001G | 450 | 28.0838 | 84.2396 | 12 months | 36.89±4.06 | 105.26±6.52 | 109.23 |
| 001H | 420 | 28.0783 | 84.238 | 1months | 50.45±1.26 | 130.91±2.08 | 75.78 |
| 001H | 420 | 28.0783 | 84.238 | 6 months | 44.42±1.51 | 119.75±4.82 | 84.08 |
| 001H | 420 | 28.0783 | 84.238 | 12 months | 35.61±0.98 | 94.75±1.8 | 111.2 |
| 001I | 845 | 28.1214 | 84.184 | 1months | 50.63±0.92 | 188.41±9.16 | 66.67 |
| 001I | 845 | 28.1214 | 84.184 | 6 months | 42.29±0.88 | 139.08±5.25 | 86.79 |
| 001I | 845 | 28.1214 | 84.184 | 12 months | 34.02±0.35 | 90.25±4.76 | 114.02 |
| 001J | 1440 | 28.2851 | 83.8508 | 1months | 72.29±1.78 | 209.58±3.81 | 63.56 |
| 001J | 1440 | 28.2851 | 83.8508 | 6 months | 58.2±1.69 | 158.08±3.32 | 90.31 |
| 001J | 1440 | 28.2851 | 83.8508 | 12 months | 37.04±0.91 | 126.75±16.09 | 116.15 |
| 001K | 1160 | 28.314 | 83.9135 | 1months | 104.56±3.26 | 362.58±7.94 | 70.32 |
| 001K | 1160 | 28.314 | 83.9135 | 6 months | 93.9±1 | 279.58±12.55 | 83.99 |
| 001K | 1160 | 28.314 | 83.9135 | 12 months | 72.33±1.9 | 193.25±5.76 | 121.44 |
| 001L | 1380 | 28.1822 | 83.6922 | 1months | 44.16±0.44 | 94.75±1.8 | 74.91 |
| 001L | 1380 | 28.1822 | 83.6922 | 6 months | 35.13±0.88 | 88.58±1.89 | 90.54 |
| 001L | 1380 | 28.1822 | 83.6922 | 12 months | 22.09±0.56 | 66.58±8.46 | 134.16 |
| 001M | 914 | 28.3426 | 83.567 | 1months | 97.45±2.33 | 231.91±9.43 | 65.07 |
| 001M | 914 | 28.3426 | 83.567 | 6 months | 67.72±2.39 | 202.55±9.92 | 83.01 |
| 001M | 914 | 28.3426 | 83.567 | 12 months | 42.42±1.72 | 157.08±3.54 | 128.36 |
| Ascorbic acid | | - | - | 1 months | - | - | 44.46 |
| Ascorbic acid | | - | - | 6 months | - | - | 45.64 |
| Ascorbic acid | | - | - | 12 months | - | - | 44.99 |

Table S1: (B) Analysis time, TPC, TFC and IC50 values of 002- *Curcuma longa*-17 samples

| **SampleID** | **Altitude(m)** | **Latitude** | **Longitude** | **Time** | **TPC(mg GAE/g) ( 3 replicate)** | **TFC (mg QE/g) ( 3 replicate)** | **IC _50_ (µg/mL)** |
| --- | --- | --- | --- | --- | --- | --- | --- |
| 002A | 1260 | 28.1095 | 83.8313 | 1months | 163.04±0.01 | 36.17±0.55 | 74.77 |
| 002A | 1260 | 28.1095 | 83.8313 | 6 months | 128.54±0.01 | 24.54±0.02 | 102.08 |
| 002A | 1260 | 28.1095 | 83.8313 | 12 months | 56.45±0.01 | 19.03±0.74 | 135.21 |
| 002B | 450 | 28.0838 | 84.2396 | 1months | 137.45±0.01 | 38.88±0.14 | 78.3 |
| 002B | 450 | 28.0838 | 84.2396 | 6 months | 98.45±0 | 27.07±0.49 | 105.33 |
| 002B | 450 | 28.0838 | 84.2396 | 12 months | 34.79±0.05 | 22.28±1.28 | 131.9 |
| 002C | 1089 | 28.1497 | 83.8584 | 1months | 141.04±0.04 | 38.63±0.89 | 64.82 |
| 002C | 1089 | 28.1497 | 83.8584 | 6 months | 113.79±0.02 | 32.05±0.3 | 110.78 |
| 002C | 1089 | 28.1497 | 83.8584 | 12 months | 39.12±0.02 | 23.26±0.47 | 143.67 |
| 002D | 420 | 28.0783 | 84.238 | 1months | 92.45±0.01 | 24.67±0.35 | 74.66 |
| 002D | 420 | 28.0783 | 84.238 | 6 months | 57.54±0 | 18.83±0.4 | 105.78 |
| 002D | 420 | 28.0783 | 84.238 | 12 months | 31.04±0.05 | 13.42±0.97 | 129.38 |
| 002E | 822 | 28.2236 | 83.9781 | 1months | 121.37±0.01 | 26.31±0.29 | 61.23 |
| 002E | 822 | 28.2236 | 83.9781 | 6 months | 88.95±0 | 22.14±0.38 | 102.89 |
| 002E | 822 | 28.2236 | 83.9781 | 12 months | 42.12±0.02 | 14±1.09 | 143.88 |
| 002F | 845 | 28.1214 | 84.184 | 1months | 64.87±0.01 | 20.09±0.19 | 77.35 |
| 002F | 845 | 28.1214 | 84.184 | 6 months | 57.7±0 | 19.48±0.32 | 113.64 |
| 002F | 845 | 28.1214 | 84.184 | 12 months | 28.7±0.02 | 15.89±0.78 | 151.19 |
| 002G | 1540 | 28.185 | 83.8446 | 1months | 44.79±0.01 | 19.48±0.23 | 71.15 |
| 002G | 1540 | 28.185 | 83.8446 | 6 months | 37.2±0.03 | 16.29±0.2 | 101.57 |
| 002G | 1540 | 28.185 | 83.8446 | 12 months | 25.04±0.01 | 11.97±0.73 | 131.22 |
| 002H | 1168 | 28.314 | 83.9135 | 1  months | 62.62±0.02 | 16.88±0.64 | 81.95 |
| 002H | 1168 | 28.314 | 83.9135 | 6 months | 49.37±0 | 16.75±0.21 | 106.91 |
| 002H | 1168 | 28.314 | 83.9135 | 12 months | 24.87±0.01 | 9.16±0.56 | 136.45 |
| 002I | 1410 | 28.1822 | 83.6922 | 1months | 115.7±0.01 | 28.75±0.62 | 68.68 |
| 002I | 1410 | 28.1822 | 83.6922 | 6 months | 81.87±0 | 20.95±0.01 | 104.08 |
| 002I | 1410 | 28.1822 | 83.6922 | 12 months | 32.45±0.02 | 7.54±0.41 | 131.21 |
| 002J | 160 | 27.7055 | 84.3888 | 1months | 64.62±0 | 18.25±0.45 | 74.37 |
| 002J | 160 | 27.7055 | 84.3888 | 6 months | 55.2±0.02 | 16.43±0.28 | 103.71 |
| 002J | 160 | 27.7055 | 84.3888 | 12 months | 28.48±0.04 | 13.53±0.88 | 143.96 |
| 002K | 890 | 28.235 | 83.6641 | 1months | 173.12±0.01 | 35.29±0.24 | 72.33 |
| 002K | 890 | 28.235 | 83.6641 | 6 months | 123.7±0 | 28.07±0.23 | 111.24 |
| 002K | 890 | 28.235 | 83.6641 | 12 months | 52.62±0.01 | 7.34±0.63 | 138.13 |
| 002L | 1103 | 28.2949 | 83.5896 | 1months | 113.45±0.02 | 30.39±0.28 | 89.23 |
| 002L | 1103 | 28.2949 | 83.5896 | 6 months | 83.7±0.01 | 29.56±0.48 | 106.53 |
| 002L | 1103 | 28.2949 | 83.5896 | 12 months | 30.95±0.02 | 20.92±1.15 | 128.53 |
| 002M | 1080 | 28.3914 | 83.5204 | 1months | 141.87±0 | 41.33±0.48 | 65.64 |
| 002M | 1080 | 28.3914 | 83.5204 | 6 months | 108.37±0.01 | 29.03±0.08 | 101.85 |
| 002M | 1080 | 28.3914 | 83.5204 | 12 months | 53.62±0.02 | 18.24±1.26 | 131.64 |
| 002N | 920 | 28.3575 | 83.5197 | 1months | 179.87±0.02 | 38.34±0.43 | 85.97 |
| 002N | 920 | 28.3575 | 83.5197 | 6 months | 148.45±0.01 | 32.35±0.26 | 111.15 |
| 002N | 920 | 28.3575 | 83.5197 | 12 months | 52.45±0.03 | 19.15±0.53 | 147.7 |
| 002O | 1540 | 28.285 | 83.8513 | 1months | 106.7±0 | 39.78±0.48 | 73.8 |
| 002O | 1540 | 28.285 | 83.8513 | 6 months | 70.45±0.01 | 38.02±0.34 | 102.09 |
| 002O | 1540 | 28.285 | 83.8513 | 12 months | 21.95±0.02 | 22.18±0.64 | 130.27 |
| 002P | 319 | 27.7006 | 84.134 | 1months | 60.95±0.01 | 18.45±0.62 | 74.2 |
| 002P | 319 | 27.7006 | 84.134 | 6 months | 56.37±0.01 | 18.25±0.36 | 113.79 |
| 002P | 319 | 27.7006 | 84.134 | 12 months | 21.54±0.04 | 9±1.65 | 158.1 |
| 002Q | 445 | 27.9738 | 84.0875 | 1months | 121.45±0.02 | 31.27±0.32 | 82.74 |
| 002Q | 445 | 27.9738 | 84.0875 | 6 months | 95.12±0.01 | 29.5±0.33 | 102.8 |
| 002Q | 445 | 27.9738 | 84.0875 | 12 months | 30.12±0.03 | 18.21±6.03 | 135.95 |
| Ascorbic acid | - | - | - | 1 months | - | - | 44.02 |
| Ascorbic acid | - | - | - | 6 months | - | - | 44.03 |
| Ascorbic acid | - | - | -- | 12 months | - | - | 45.89 |

Table S1: (C). Analysis time, TPC, TFC and IC50 values of 003- *Zanthoxylum armatum*-7 samples

| **SampleID** | **Altitude(m)** | **Latitude** | **Longitude** | **Time** | **TPC(mg GAE/g) ( 3 replicate)** | **TFC (mg QE/g) ( 3 replicate)** | **IC _50_ (µg/mL)** |
| --- | --- | --- | --- | --- | --- | --- | --- |
| 003A | 1610 | 28.1095 | 83.8313 | 1months | 214.05±3.59 | 93.78±1 | 78.87 |
| 003A | 1610 | 28.1095 | 83.8313 | 6 months | 118.29±1.45 | 67.25±2.29 | 96.16 |
| 003A | 1610 | 28.1095 | 83.8313 | 12 months | 63.1±1.79 | 43.26±1.71 | 123.65 |
| 003B | 1120 | 28.3752 | 83.5433 | 1months | 235.57±2.83 | 114.58±2.92 | 62.7 |
| 003B | 1120 | 28.3752 | 83.5433 | 6 months | 126.89±3.84 | 85.08±1.04 | 78.02 |
| 003B | 1120 | 28.3752 | 83.5433 | 12 months | 92.59±2.48 | 54.75±3.04 | 106.6 |
| 003C | 1240 | 28.5255 | 83.5168 | 1months | 240.72±2.35 | 146.58±3.4 | 65.1 |
| 003C | 1240 | 28.5255 | 83.5168 | 6 months | 129.24±1.91 | 101.91±0.57 | 80.46 |
| 003C | 1240 | 28.5255 | 83.5168 | 12 months | 102.23±1.53 | 72.08±2.08 | 105.38 |
| 003D | 920 | 28.2321 | 83.8996 | 1months | 206.65±0.32 | 115.41±2.08 | 70.24 |
| 003D | 920 | 28.2321 | 83.8996 | 6 months | 120.27±0.16 | 78.25±5.56 | 85.54 |
| 003D | 920 | 28.2321 | 83.8996 | 12 months | 91.45±0.07 | 41.11±0.5 | 131.93 |
| 003E | 2420 | 28.5511 | 84.237 | 1months | 230.26±1.19 | 146.75±4.35 | 66.24 |
| 003E | 2420 | 28.5511 | 84.237 | 6 months | 110.52±3.59 | 103.58±0.76 | 88.14 |
| 003E | 2420 | 28.5511 | 84.237 | 12 months | 93.87±2.11 | 60.25±4.58 | 101.49 |
| 003F | 2100 | 28.2908 | 84.3032 | 1months | 216.85±3.85 | 121.41±0.57 | 62.97 |
| 003F | 2100 | 28.2908 | 84.3032 | 6 months | 125.45±8.49 | 86.91±3.4 | 78.38 |
| 003F | 2100 | 28.2908 | 84.3032 | 12 months | 93.89±0.74 | 47.95±2.55 | 145.05 |
| 003G | 1876 | 28. 393820 | 83.197 | 1months | 239.75±2.81 | 114.91±2.08 | 66.77 |
| 003G | 1876 | 28. 393823 | 83.197 | 6 months | 113.66±2.43 | 91.76±2.5 | 81.99 |
| 003G | 1876 | 28. 393826 | 83.197 | 12 months | 91.28±3.65 | 44.49±0.75 | 126.4 |
| Ascorbic acid | - | - | - | 1 months | - | - | 40.35 |
| Ascorbic acid | - | - | - | 6 months | - | - | 43.16 |
| Ascorbic acid | - | - | - | 12 months | - | - | 43.92 |

Table S2: (A)-1. Analysis time and I% value of 001- *Cinamomum tamala*-13 samples (1st month-1^st^ Analysis)

| **Concentration(µg/ml)** | **I%-Asc** | **I%-001A** | **I%-001B** | **I%-001C** | **I%-001D** | **I%-001E** | **I%-001F** | **I%-001G** | **I%-001H** | **I%-001I** | **I%-001J** | **I%-001K** | **I%-001L** | **I%-001M** |
| --- | --- | --- | --- | --- | --- | --- | --- | --- | --- | --- | --- | --- | --- | --- |
| 0 | 0 | 0 | 0 | 0 | 0 | 0 | 0 | 0 | 0 | 0 | 0 | 0 | 0 | 0 |
| 25 | 43.0032 | 30.012 | 35.8009 | 32.055 | 29.78 | 28.789 | 31.552 | 27.5234 | 26.8 | 36.1254 | 33.55678 | 31.0158 | 29.5552 | 33.551 |
| 50 | 68.5679 | 45.9167 | 48.9231 | 47.554 | 46.8008 | 43.1276 | 46.58 | 50.1005 | 41.947 | 48.556 | 47.02145 | 46.451 | 38.8452 | 50.1108 |
| 75 | 83.5752 | 58.905 | 60.123 | 57.887 | 69.001 | 59.099 | 55.008 | 67.008 | 53.948 | 59.5177 | 69.458 | 58.4757 | 63.051 | 62.0445 |
| 100 | 90.52225 | 70.18468 | 71.105 | 66.3544 | 70.201 | 67.8579 | 64.748 | 82.1453 | 69.251 | 76.005 | 77.459 | 73.4508 | 68.735 | 77.2558 |
| 125 | 94.35201 | 79.5092 | 84.1981 | 69.9751 | 79.118 | 77.0021 | 72.5 | 86.25 | 75.245 | 78.518 | 79.2591 | 77.155 | 72.1215 | 81.5114 |
| 150 | 96.05455 | 80.189 | 85.5585 | 80.5451 | 80.2655 | 78.2349 | 74.55 | 88 | 79.954 | 80.671 | 87.039 | 80.2585 | 78.0046 | 82.1982 |
| IC50 (µg/ml) | 44.46 | 70.95 | 65.49 | 73.699 | 68.06 | 73.77 | 76.58 | 62.65 | 75.78 | 66.67 | 63.56 | 70.32 | 74.91 | 65.07 |

Table S2: (A)-2. Analysis time and I% value of 001- *Cinamomum tamala*-13 samples (6^th^ month-2^nd^ Analysis)

| **Concentration(µg/ml)** | **I%-Asc** | **I%-001A** | **I%-001B** | **I%-001C** | **I%-001D** | **I%-001E** | **I%-001F** | **I%-001G** | **I%-001H** | **I%-001I** | **I%-001J** | **I%-001K** | **I%-001L** | **I%-001M** |
| --- | --- | --- | --- | --- | --- | --- | --- | --- | --- | --- | --- | --- | --- | --- |
| 0 | 0 | 0 | 0 | 0 | 0 | 0 | 0 | 0 | 0 | 0 | 0 | 0 | 0 | 0 |
| 25 | 53.009 | 22.25 | 22.5047 | 24.0056 | 27.125 | 25.5154 | 22.001 | 23.19 | 22.454 | 30.097 | 29.15401 | 28.599 | 27.008 | 30.005 |
| 50 | 60.44558 | 35.251 | 43.002 | 34.807 | 42.9871 | 40.2525 | 37.55 | 44.999 | 40.0056 | 45.7145 | 34.0148 | 46.5345 | 22.1233 | 35.4445 |
| 75 | 78.01447 | 49.0018 | 57.1256 | 43.1552 | 51.505 | 59.81559 | 49.8801 | 54.1205 | 51.845 | 50.7413 | 43.2441 | 56.5022 | 51.3213 | 43.1059 |
| 100 | 87.1185 | 69.5004 | 64.2551 | 49.5404 | 64.598 | 62.8732 | 51.0012 | 63.0092 | 63.3231 | 54.998 | 49.6005 | 58.0015 | 56.8979 | 63.7142 |
| 125 | 89.544 | 71.245 | 68.5102 | 69.0111 | 67.551 | 68.254 | 71.0554 | 70.845 | 67.8254 | 59.007 | 69.00145 | 63.554 | 61.1123 | 71.4044 |
| 150 | 93.5 | 73.008 | 70.98 | 70.9559 | 73.005 | 71.051 | 73.5255 | 73.423 | 74.003 | 75.1003 | 75.645 | 70.5101 | 73.018 | 78.554 |
| IC50 (µg/ml) | 45.64 | 83.47 | 82.07 | 92.46 | 82.21 | 81.92 | 88.54 | 81.11 | 84.08 | 86.79 | 90.31 | 83.99 | 90.54 | 83.01 |

Table S2: (A)-3 Analysis time and I% value of 001- *Cinamomum tamala*-13 samples (12^th^ month-3^rd^ Analysis)

| **Concentration(µg/ml)** | **I%-Asc** | **I%-001A** | **I%-001B** | **I%-001C** | **I%-001D** | **I%-001E** | **I%-001F** | **I%-001G** | **I%-001H** | **I%-001I** | **I%-001J** | **I%-001K** | **I%-001L** | **I%-001M** |
| --- | --- | --- | --- | --- | --- | --- | --- | --- | --- | --- | --- | --- | --- | --- |
| 0 | 0 | 0 | 0 | 0 | 0 | 0 | 0 | 0 | 0 | 0 | 0 | 0 | 0 | 0 |
| 25 | 50.0145 | 9.345 | 12.155 | 7.456 | 10.014 | 13.885 | 18.6577 | 16.2547 | 9.9907 | 17.4565 | 11.854 | 12.001 | 9.544 | 9.0045 |
| 50 | 62.1211 | 17.52344 | 24.997 | 10.995 | 22.4771 | 19.977 | 31.0055 | 29.0989 | 19.0045 | 31.578 | 21.544 | 23.2005 | 20.045 | 17.6405 |
| 75 | 79.5121 | 26.5155 | 34.0025 | 22.5098 | 32.5502 | 28.5678 | 33.599 | 41.705 | 30.587 | 33.5545 | 34.854 | 34.4784 | 31.0256 | 31.0255 |
| 100 | 89.006 | 43.9947 | 56.5475 | 44.8752 | 60.477 | 49.0048 | 42.1147 | 50.0875 | 54.987 | 51.5155 | 48.9801 | 40.254 | 35.4541 | 45.4141 |
| 125 | 94.1398 | 65.0047 | 65.4858 | 49.5123 | 61.5545 | 55.0063 | 59.025 | 57.6565 | 59.235 | 54.556 | 56.0014 | 56.3253 | 50.5217 | 50.257 |
| 150 | 97.654 | 68.105 | 66.14 | 54.8814 | 63.984 | 57.7605 | 60.654 | 59.0466 | 61.1002 | 57.402 | 57.789 | 56.259 | 52.555 | 52.984 |
| IC50 (µg/ml) | 44.99 | 109.93 | 101.93 | 131.51 | 104.7 | 119.21 | 113.31 | 109.23 | 111.2 | 114.02 | 116.15 | 121.44 | 134.16 | 128.36 |

Table S2: (B)-1. Analysis time and I% value of 002- *Curcuma longa* -17 samples (1^st^ month-1^st^ Analysis)

| **Concentration(µg/ml)** | **I%-Asc** | **I%-002A** | **I%-002B** | **I%-002C** | **I%-002D** | **I%-002E** | **I%-002F** | **I%-002G** | **I%-002H** | **I%-002I** | **I%-002J** | **I%-002K** | **I%-002L** | **I%-002M** | **I%-002N** | **I%-002O** | **I%-002P** | **I%-002Q** |
| --- | --- | --- | --- | --- | --- | --- | --- | --- | --- | --- | --- | --- | --- | --- | --- | --- | --- | --- |
| 0 | 0 | 0 | 0 | 0 | 0 | 0 | 0 | 0 | 0 | 0 | 0 | 0 | 0 | 0 | 0 | 0 | 0 | 0 |
| 25 | 47.7585 | 26.505 | 29.558 | 32.005 | 32.5001 | 37.567 | 24.975 | 29.977 | 29.0047 | 33.3422 | 25.988 | 30.334 | 20.987 | 35.5 | 29.498 | 27.3435 | 27.95 | 30.144 |
| 50 | 60.6896 | 49.8004 | 37.2258 | 46.5002 | 44.9005 | 49.8231 | 39.2345 | 44.789 | 41.9342 | 47.3418 | 40.878 | 44.1455 | 34.125 | 48.159 | 38.125 | 42.232 | 42.87 | 42.74 |
| 75 | 69.6551 | 53.007 | 59.007 | 68.8134 | 58.1159 | 66.5453 | 57.885 | 60.6538 | 56.3346 | 59.005 | 59.3213 | 57.0054 | 46.478 | 60.6709 | 45.5 | 54.1225 | 54.116 | 52.115 |
| 100 | 86.5517 | 63.801 | 61.9855 | 76.986 | 67.665 | 79.3389 | 69.3452 | 68.5569 | 60.8777 | 70.906 | 68.0127 | 69.985 | 63.851 | 76.128 | 59.875 | 68.1435 | 70.885 | 61.045 |
| 125 | 89.3103 | 74.8112 | 70.855 | 79.5456 | 71.9235 | 81.876 | 72.0028 | 76.656 | 67.1245 | 79.9763 | 77.1208 | 78.0254 | 66.1521 | 79.899 | 69.985 | 79.567 | 76.987 | 68.9875 |
| 150 | 90.3448 | 82.881 | 79.8752 | 84.7665 | 76.008 | 87.0568 | 78.126 | 83.511 | 72.905 | 82.4612 | 81.0003 | 80.1325 | 71.009 | 84.212 | 72.0078 | 83.1349 | 80.1215 | 71.045 |
| IC-50 value(µg/ml) | 44.02 | 74.77 | 78.3 | 64.82 | 74.66 | 61.23 | 77.35 | 71.15 | 81.95 | 68.68 | 74.37 | 72.33 | 89.23 | 65.64 | 85.97 | 73.8 | 74.2 | 82.74 |

Table S2: (B)-2. Analysis time and I% value of 002- *Curcuma longa* -17 samples (6^th^ month-2^nd^ Analysis)

| **Concentration(µg/ml)** | **I%-Asc** | **I%-002A** | **I%-002B** | **I%-002C** | **I%-002D** | **I%-002E** | **I%-002F** | **I%-002G** | **I%-002H** | **I%-002I** | **I%-002J** | **I%-002K** | **I%-002L** | **I%-002M** | **I%-002N** | **I%-002O** | **I%-002P** | **I%-002Q** |
| --- | --- | --- | --- | --- | --- | --- | --- | --- | --- | --- | --- | --- | --- | --- | --- | --- | --- | --- |
| 0 | 0 | 0 | 0 | 0 | 0 | 0 | 0 | 0 | 0 | 0 | 0 | 0 | 0 | 0 | 0 | 0 | 0 | 0 |
| 25 | 47.78 | 19.11 | 25.865 | 19.15 | 23.156 | 25.243 | 21.366 | 16.995 | 22.02 | 27.56 | 24.01 | 18.95 | 23.49 | 20.125 | 21.598 | 20.003 | 21.07 | 20.11 |
| 50 | 62.1 | 25.115 | 28.558 | 24.511 | 35.987 | 32.3223 | 32.98 | 23.5 | 34.874 | 31.09 | 36.5 | 23.27 | 35.62 | 38.435 | 33.002 | 35.36 | 32.88 | 37.1212 |
| 75 | 85.33 | 36.32 | 38.9365 | 30.328 | 42.658 | 52.153 | 39.54 | 47.98 | 41.98 | 39.011 | 43.1225 | 30.105 | 42.92 | 45.85 | 40.41 | 51.145 | 39.39 | 44.65 |
| 100 | 91.15 | 56.44 | 52.21 | 42.95 | 49.977 | 52.898 | 46.32 | 54.887 | 48.574 | 43.22 | 50.115 | 52.978 | 49.05 | 52.5 | 47.75 | 54.245 | 46.785 | 51.66 |
| 125 | 95.01 | 62.98 | 58.145 | 60.23 | 56.688 | 56.3001 | 52.918 | 61.894 | 55.984 | 63.199 | 57 | 59.15 | 56.22 | 59.09 | 53.654 | 57.557 | 52.5 | 58.9 |
| 150 | 96.8 | 64.9756 | 62.568 | 66.005 | 60.585 | 58.8143 | 58.99 | 62.654 | 61.574 | 64.981 | 62.532 | 60.198 | 60.543 | 60.544 | 59.99 | 58.59 | 58.98 | 61.05 |
| IC-50 value(µg/ml) | 44.03 | 102.08 | 105.33 | 110.78 | 105.78 | 102.89 | 113.64 | 101.57 | 106.91 | 104.08 | 103.71 | 111.24 | 106.53 | 101.85 | 111.15 | 102.09 | 113.79 | 102.8 |

Table S2: (B)-3. Analysis time and I% value of 002- *Curcuma longa* -17 samples (12^th^ month-3^rd^ Analysis)

| **Concentration(µg/ml)** | **I%-Asc** | **I%-002A** | **I%-002B** | **I%-002C** | **I%-002D** | **I%-002E** | **I%-002F** | **I%-002G** | **I%-002H** | **I%-002I** | **I%-002J** | **I%-002K** | **I%-002L** | **I%-002M** | **I%-002N** | **I%-002O** | **I%-002P** | **I%-002Q** |
| --- | --- | --- | --- | --- | --- | --- | --- | --- | --- | --- | --- | --- | --- | --- | --- | --- | --- | --- |
| 0 | 0 | 0 | 0 | 0 | 0 | 0 | 0 | 0 | 0 | 0 | 0 | 0 | 0 | 0 | 0 | 0 | 0 | 0 |
| 25 | 49.89 | 9.98 | 9.015 | 15.005 | 10.02 | 13.98 | 11.76 | 13.44 | 9.99 | 10.08 | 15.65 | 11.323 | 12.115 | 14.44 | 9.21 | 7.95 | 15.22 | 10.45 |
| 50 | 62.148 | 20.214 | 15.258 | 16.66 | 16.62 | 16.874 | 14.88 | 17.051 | 11.087 | 18.77 | 19.07 | 16.543 | 17.015 | 18.93 | 13.46 | 18.01 | 19.35 | 13.235 |
| 75 | 76.5 | 22.851 | 19.895 | 22.122 | 22.98 | 22.772 | 20.421 | 24.66 | 22.54 | 26.26 | 24.23 | 22.98 | 25.67 | 23.215 | 18.67 | 23.47 | 26.32 | 24.33 |
| 100 | 88.558 | 39.88 | 29.81 | 26.98 | 43.43 | 37.544 | 26.55 | 42.089 | 40.145 | 47.461 | 26.75 | 29.02 | 48.97 | 42.214 | 40.44 | 46.221 | 29.78 | 43.7 |
| 125 | 94.15 | 50.5 | 54.5 | 50.98 | 46.225 | 45.112 | 40.125 | 49.9 | 47.75 | 49.0433 | 49.65 | 53.15 | 50.55 | 49.5 | 45.82 | 50.71 | 42.04 | 47.47 |
| 150 | 96.2 | 51.99 | 59.66 | 52.877 | 59.588 | 51.05 | 56.05 | 55.021 | 54.22 | 51.55 | 52.95 | 54.015 | 52.75 | 55.125 | 46.995 | 52.94 | 46.97 | 52.012 |
| IC-50 value(µg/ml) | 45.89 | 135.21 | 131.9 | 143.67 | 129.38 | 143.88 | 151.19 | 131.22 | 136.45 | 131.21 | 143.96 | 138.13 | 128.53 | 131.64 | 147.7 | 130.27 | 158.1 | 135.95 |

Table S2: (C)-1. Analysis Time and I% value of 003-*Zanthoxylum armatum*-7 samples (1st month-1^st^ Analysis)

| **Concentration(µg/ml)** | **I%-Asc** | **I%-003A** | **I%-003B** | **I%-003C** | **I%-003D** | **I%-003E** | **I%-003F** | **I%-003G** |
| --- | --- | --- | --- | --- | --- | --- | --- | --- |
| 0 | 0 | 0 | 0 | 0 | 0 | 0 | 0 | 0 |
| 25 | 57.7204 | 43.769 | 40.345 | 42.158 | 36.5519 | 40.0002 | 31.3795 | 33.4485 |
| 50 | 70.082 | 44.9848 | 53.414 | 54.4073 | 58.6553 | 54.1381 | 57.5864 | 46.2071 |
| 75 | 78.5623 | 48.0243 | 62.4139 | 61.3982 | 60.345 | 61.6898 | 66.8967 | 70.3449 |
| 100 | 85.1185 | 55.0152 | 71.3794 | 67.7811 | 64.1381 | 71.9311 | 76.207 | 73.4484 |
| 125 | 89.7143 | 55.927 | 82.069 | 76.5957 | 67.9311 | 74.138 | 78.9656 | 74.8277 |
| 150 | 95.5045 | 60.7903 | 83.7242 | 79.3313 | 76.5518 | 75.5173 | 82.4139 | 80.6897 |
| IC50 (µg/ml) | 40.35 | 78.87 | 62.7 | 65.1 | 70.24 | 66.24 | 62.97 | 66.77 |

Table S2: (C)-2. Analysis time and I% value of 003-*Zanthoxylum armatum*-7 samples (6^th^ month-2^nd^ Analysis)

| **Concentration(µg/ml)** | **I%-Asc** | **I%-003A** | **I%-003B** | **I%-003C** | **I%-003D** | **I%-003E** | **I%-003F** | **I%-003G** |
| --- | --- | --- | --- | --- | --- | --- | --- | --- |
| 0 | 0 | 0 | 0 | 0 | 0 | 0 | 0 | 0 |
| 25 | 54.0457 | 18.7298 | 28.564 | 17.8503 | 22.3063 | 33.3502 | 26.6614 | 22.14 |
| 50 | 68.2855 | 24.593 | 44.635 | 43.5343 | 34.4922 | 45.585 | 43.0164 | 46.4822 |
| 75 | 77.4767 | 56.0261 | 57.897 | 58.8926 | 44.075 | 49.6222 | 51.4659 | 58.3063 |
| 100 | 83.7134 | 57.9219 | 78.662 | 64.4365 | 63.1924 | 52.85 | 58.9903 | 63.2521 |
| 125 | 90.0652 | 62.1661 | 80.9235 | 67.5766 | 72.0847 | 65.5426 | 76.5049 | 66.645 |
| 150 | 94.6938 | 64.2997 | 81.5788 | 78.329 | 76.2476 | 67.1544 | 81.2085 | 70.8795 |
| IC50 (µg/ml) | 43.16 | 96.16 | 78.02 | 80.46 | 85.54 | 88.14 | 78.38 | 81.99 |

Table S2: (C)-3. Analysis time and I% value of 003-*Zanthoxylum armatum*-7 samples (12th month-3^rd^ Analysis)

| **Concentration(µg/ml)** | **I%-Asc** | **I%-003A** | **I%-003B** | **I%-003C** | **I%-003D** | **I%-003E** | **I%-003F** | **I%-003G** |
| --- | --- | --- | --- | --- | --- | --- | --- | --- |
| 0 | 0 | 0 | 0 | 0 | 0 | 0 | 0 | 0 |
| 25 | 55.2035 | 16.8863 | 20.4057 | 11.0665 | 15.0099 | 19.467 | 19.0456 | 20.022 |
| 50 | 69.5191 | 19.3663 | 34.5556 | 23.652 | 23.097 | 35.755 | 34.3451 | 34.4564 |
| 75 | 76.1908 | 36.9763 | 43.562 | 34.433 | 33.997 | 45.6678 | 38.9607 | 41.0046 |
| 100 | 78.6985 | 46.442 | 48.3467 | 56.567 | 45.6778 | 56.5564 | 40.4333 | 45.4756 |
| 125 | 85.7143 | 47.7376 | 58.086 | 61.3324 | 47.0099 | 58.607 | 42.3946 | 47.3575 |
| 150 | 95.5045 | 56.6636 | 60.0021 | 64.045 | 50.2 | 60.0357 | 44.4564 | 50.3257 |
| IC50 (µg/ml) | 43.92 | 123.65 | 106.6 | 105.38 | 131.93 | 101.49 | 145.05 | 126.4 |

Table S3: Time and variable (phytochemical and antioxidant bioactivities)- p value

| **S.N** | **Sample Name** | **Variable** | **P (ANOVA)** |
| --- | --- | --- | --- |
| 1 | *C. tamala*-TPC | TPC | 5.60E-12 |
| 2 | *C. tamala* -TFC | TFC | 2.78E-08 |
| 3 | *C. tamala* -IC50 | IC50 | 1.31E-18 |
| 4 | *C. longa*-TPC | TPC | 6.13E-25 |
| 5 | *C. longa* -TFC | TFC | 2.85E-18 |
| 6 | *C. longa* -IC50 | IC50 | 3.06E-29 |
| 7 | *Z. armatum* -TPC | TPC | 3.71E-45 |
| 8 | *Z. armatum* -TFC | TFC | 2.02E-22 |
| 9 | *Z. armatum* -IC50 | IC50 | 9.24E-08 |

* Time has a significant effect on TPC, TFC and IC50. p-values <0.05.

Table S4: Time and variable (phytochemical and antioxidant bioactivities) - Group wise Tukey HSD summary

| **S.n** | **Sample Name** | **Group1** | **Group2** | **Variable** | **Reject** | **p_adj** |
| --- | --- | --- | --- | --- | --- | --- |
| 1 | *C. tamala* -TPC | 1 month | 12 months | TPC | TRUE | 0 |
| 2 | *C. tamala* -TPC | 1 month | 6 months | TPC | TRUE | 0.0024 |
| 3 | *C. tamala* -TPC | 12 months | 6 months | TPC | TRUE | 0 |
| 4 | *C. tamala* -TFC | 1 month | 12 months | TFC | TRUE | 0 |
| 5 | *C. tamala* -TFC | 1 month | 6 months | TFC | TRUE | 0.0004 |
| 6 | *C. tamala* -TFC | 12 months | 6 months | TFC | FALSE | 0.0551 |
| 7 | *C. tamala* -IC50 | 1 month | 12 months | IC50 | TRUE | 0 |
| 8 | *C. tamala* -IC50 | 1 month | 6 months | IC50 | TRUE | 0 |
| 9 | *C. tamala* -IC50 | 12 months | 6 months | IC50 | TRUE | 0 |
| 10 | *C. longa* -TPC | 1 month | 12 months | TPC | TRUE | 0 |
| 11 | *C. longa* -TPC | 1 month | 6 months | TPC | TRUE | 0.0001 |
| 12 | *C. longa* -TPC | 12 months | 6 months | TPC | TRUE | 0 |
| 13 | *C. longa* -TFC | 1 month | 12 months | TFC | TRUE | 0 |
| 14 | *C. longa* -TFC | 1 month | 6 months | TFC | TRUE | 0.0013 |
| 15 | *C. longa* -TFC | 1 months | 6 months | TFC | TRUE | 0 |
| 16 | *C. longa* -IC50 | 1 month | 12 months | IC50 | TRUE | 0 |
| 17 | *C. longa* -IC50 | 1 month | 6 months | IC50 | TRUE | 0 |
| 18 | *C. longa* -IC50 | 12 months | 6 months | IC50 | TRUE | 0 |
| 19 | *Z. armatum* -TPC | 1 month | 12 months | TPC | TRUE | 0 |
| 20 | *Z. armatum* -TPC | 1 month | 6 months | TPC | TRUE | 0 |
| 21 | *Z. armatum* -TPC | 12 months | 6 months | TPC | TRUE | 0 |
| 22 | *Z. armatum* -TFC | 1 month | 12 months | TFC | TRUE | 0 |
| 23 | *Z. armatum* -TFC | 1 month | 6 months | TFC | TRUE | 0 |
| 24 | *Z. armatum* -TFC | 12 months | 6 months | TFC | TRUE | 0 |
| 25 | *Z. armatum* -IC50 | 1 month | 12 months | IC50 | TRUE | 0 |
| 26 | *Z. armatum* -IC50 | 1 month | 6 months | IC50 | TRUE | 0.0228 |
| 27 | *Z. armatum* -IC50 | 12 months | 6 months | IC50 | TRUE | 0 |

* In group wise Tukey we see all groups are different except for *C. tamala*-TFC where 6 months and 12 months are not significantly different.
